# Efficacy of ginseng supplements on disease-related fatigue: A systematic review and meta-analysis

**DOI:** 10.1097/MD.0000000000029767

**Published:** 2022-06-30

**Authors:** Jianxun Zhu, Xiaoru Xu, Xin Zhang, Yue Zhuo, Shaotao Chen, Chongwen Zhong, Mingjun Liu, Zhihong Wang

**Affiliations:** a Changchun University of Chinese Medicine, Jingyue Economic Development District, Changchun, People’s Republic of China.

**Keywords:** disease-related, fatigue, ginseng supplements, meta-analysis

## Abstract

Ginseng has been believed to provide energy, physical health, and well-being to patients for hundreds of years. Fatigue is a multidimensional symptom with unknown etiology and varying severity, and lots of patients suffer from fatigue.

**Methods:**

We search for research of ginseng treatment of disease-related fatigue in adult patients in Pubmed, Embase, Medline, and Cochrane library. Two independent reviewers assessed included studies and met to develop consensus on included studies. And we used Review Manager 5.3 software to evaluate the risk of bias.

**Results:**

The present meta-analysis included 12 randomized controlled trial containing 1298 patients. In the fixed-effect meta-analysis of 12 randomized controlled trial, ginseng supplements had a statistically significant efficacy on disease-related fatigue reduction (standardized mean difference = 0.33, 95% confidence interval = 0.44–0.22).

**Conclusions:**

The use of ginseng supplements is benefit for patients to reduce disease-related fatigue.

## 1. Introduction

Fatigue is a physiological phenomenon that often occurs in the human body. Human with fatigue are characterized by low mood, low vitality, boredom, and reduced sleep; severe fatigued people will also have muscle pain, muscle weakness, and weight loss.^[1,2]^ Disease-related fatigue, a type of fatigue, is a main feeling of fatigue caused by the disease itself or its treatment, and cancer-related fatigue is the most common.^[3,4]^ Disease-related fatigue is an accompanying symptom and also seriously affects the patient’s compliance with treatment.^[5,6]^ For fatigue reduction, most patients are given alternative and complementary, including scientific sports training, nutritional supplements, Chinese medicine supplements, etc.^[7, 8]^

Ginseng, a kind of medicine or health food widely used to treat fatigue in the world, has 2 major subgroup, Korean ginseng and American ginseng. Ginseng is the dried root of the Araliaceae plant ginseng, a traditional nourishing and health-preserving precious medicinal material in China, which has the functions of invigorating vitality, rejuvenating the pulse, replenishing the spleen and lungs, nourishing body fluid, nourishing blood, and soothing mind.^[9,10]^ According to the published survey report, Korean ginseng and its extract are being accepted by consumers in Western countries for strengthening immunity and reducing fatigue.^[11]^ Because of the wild use of ginseng, more and more scholars are paying attention to the efficacy of ginseng as a treatment drug and the effect of eliminating fatigue as a nutritional supplement. To date, about 40 kinds of active substances from ginseng have been found to have a variety of biological activities, such as antioxidative stress, immune regulation, antitumor activity, antiradiation, antiaging, etc.^[12,13]^ However, these effects of ginsengs lack sufficient clinical research support.

Recently, some randomized controlled trials (RCTs) about the efficacy of ginseng supplements on disease-related fatigue have been published, but the conclusion is still unclear. And the topic of the efficacy of ginseng supplements on disease-related fatigue is still very few. In the present study, we investigated the efficacy of ginseng supplements on disease-related fatigue using a meta-analysis.

## 2. Methods

### 2.1. Ethics statement

As a study of systematic review and meta-analysis, the present study did not recruit any volunteers and did not conduct any human or animal experiments, so this study does not require ethical approval.

### 2.2. Search strategy

In January 2021, we searched for studies related to the effect of ginseng supplements on disease-related fatigue. The language is limited to English, and time is from January 2010 to December 2020. Two reviewers independently selected trials and extracted data according to predetermined selection criteria. Any inconsistencies will eventually be resolved through mutual discussion. Key words for literature search are as follow: “American Ginseng”, “Asian Ginseng”, “Panax ginseng”, “Korean ginseng”, “Korean red ginseng”, “Panax ginseng”, “fatigue”, “weariness”, “tiredness”, “exhaustion”.

### 2.3. Selection criteria

Studies included in the present meta-analysis are related to the effect of ginseng supplements on disease-related fatigue, so the studies meet the following criteria: participants with underlying diseases, RCT, any type of ginseng supplement as an intervention, placebo as a control intervention, and fatigue evaluation plan is clear.

### 2.4. Data extraction

Two reviewers independently extracted the following key data by reading the full text: author, publication date, methods of randomization, description of randomization, methods of blind, participant characteristics (gender, disease, and region), sample size, ginseng category, intervention plan, fatigue evaluation program, and outcome of fatigue evaluation.

### 2.5. Statistical analysis and heterogeneity

Review Manager 5.3 software was used for meta-analysis. We investigated the difference in fatigue reduction between placebo intervention group and ginseng supplement intervention group in patients. Due to the different scales for fatigue reduction across the studies, we used standardized mean difference (SMD) as a main effect size to calculate those differences. Each effect size is given its point estimate and 95% confidence interval (CI). If there is no statistical heterogeneity (*P* > .1), use fixed-effects model analysis. If there is heterogeneity (*P* < .1), analyze the source of heterogeneity first. If there is no obvious clinical heterogeneity and no definite source of statistical heterogeneity can be found, random-effects model analysis can be used; if there is obvious clinical heterogeneity or methodological heterogeneity or incomplete data provided, then perform descriptive analysis. If there is significant statistical heterogeneity due to the different methodological quality of the included studies, low-quality studies can be removed for sensitivity analysis.

## 3. Results

### 3.1. Selection of trials

**Figure 1.**
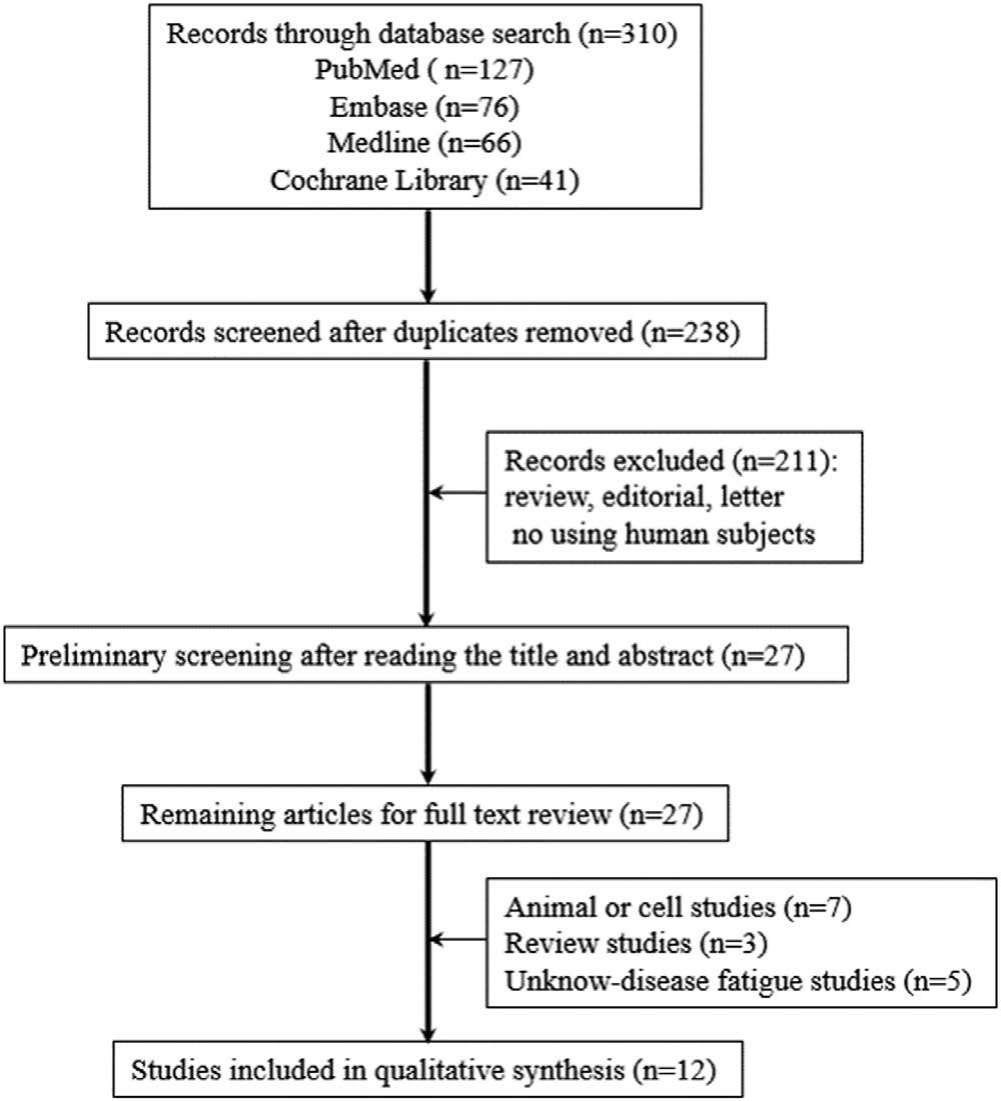
Flow diagram for identification of relevant clinical trials.

There were 310 studies found after searching in 4 databases (Fig. 1). After removing duplicate literature, 238 studies were included. Next, 2 reviewers selected 27 studies after reading the title and abstract of these trials. At last, we included 12 studies in final analysis after reading full text of 27 studies, and excluded 13 studies because of selection criteria.

### 3.2. Trials quality evaluation

**Figure 2.**
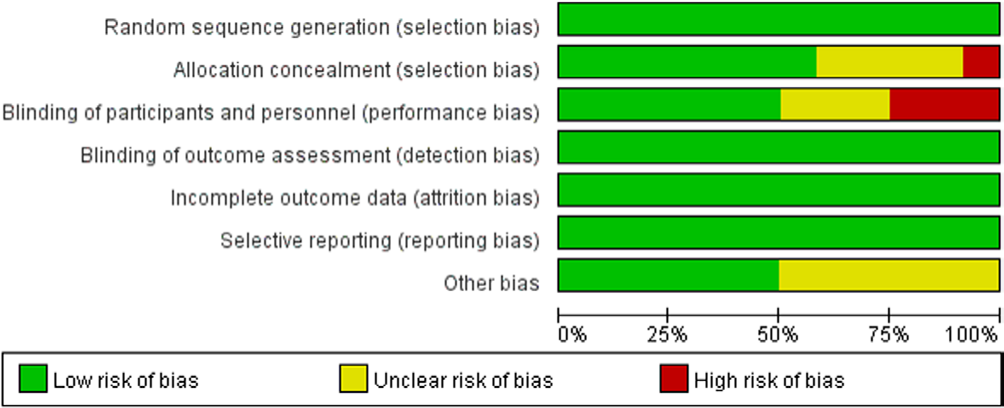
Risk of bias graph: review authors’ judgments about each risk of bias item presented as percentages across all included studies.

**Figure 3.**
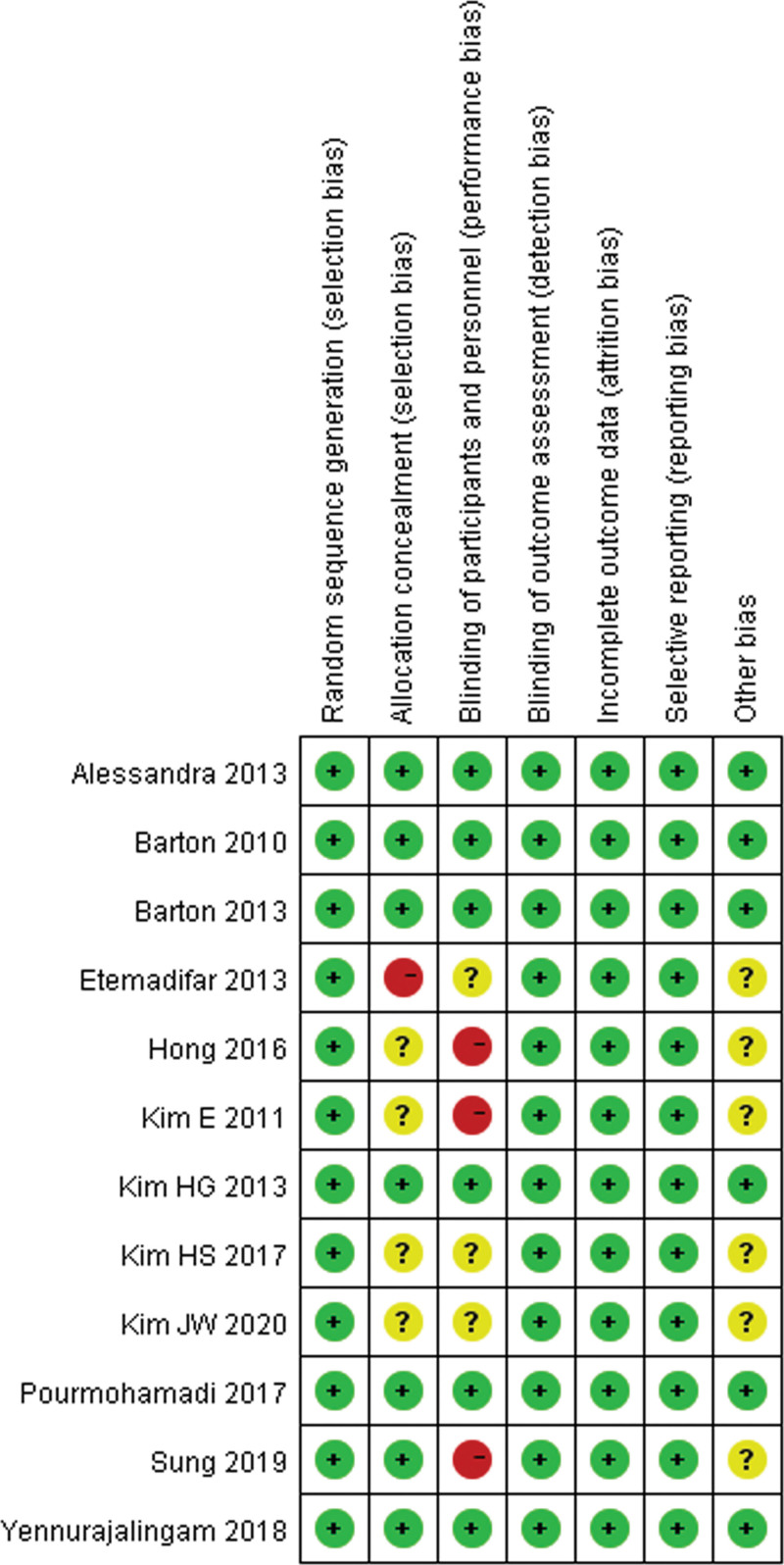
Risk of bias summary: review authors’ judgments about each risk of bias item for each included study.

Jadad scale was used to assess the methodological quality of these studies in the present meta-analysis from 5 dimensions,^[14]^ including randomization, description of randomization methods, double-blind, using identical placebo, and follow-up reporting. As shown in Table 1, all included studies were scored 3 to 5, so the study included in the present meta-analysis was low risk of bias. In addition, Review Manager 5.3 software was also used for trials’ quality: the risk of bias graph (Fig. 2) and risk of bias summary (Fig. 3).

**Table 1:** General characteristics of studies in the final analysis (n = 12).

Study (author, year)	Randomization	Description of randomization methods	Double blind	Using identical placebo	Follow-up reporting	Total score
Barton et al, 2010^[15]^	1	1	1	1	1	5
Kim et al, 2011^[16]^	1	1	1	1	1	5
Barton et al. 2013^[17]^	1	1	1	1	1	5
Kim et al, 2013^[18]^	1	1	1	0	1	4
Etemadifar et al, 2013^[19]^	1	1	1	1	1	5
Braz et al, 2013^[20]^	1	1	1	1	1	5
Hong et al, 2016^21^	1	1	1	1	1	5
Hee et al, 2017^[22]^	1	1	1	1	1	5
Pourmohamadi et al, 2017^[23]^	1	1	1	1	1	5
Yennurajalingam et al, 2018^[24]^	1	1	1	1	1	5
Sung et al, 2019^25^	1	1	1	1	0	4
Kim et al, 2020^26^	1	1	1	1	1	5

### 3.3. Characteristics s of included trails

General characteristics of studies (n = 12) in the final analysis are shown in Table 2. A total of 1298 participants were included in final trials, and the dose of ginseng supplement intervention ranged from 100 to 3000 mg/d, and the duration from 3 to 16 weeks. The area where the trials were located included United States (n = 4), South Korea (n = 5), Iran (n=2), and Brazil (n = 1). The type of ginseng included American ginseng (n = 5) and Asian ginseng (n = 7). The tools for evaluating fatigue are so many, including Brief Fatigue Inventory (BFI), Fatigue Severity Scale, Real-Time Digital Fatigue Score (RDFS), Modified Fatigue Impact Scale, Multidimensional Fatigue Symptom Inventory-Short Form(MFSI-SF), numerical rating scale, visual analog scale, Multiple Sclerosis Quality of Life Questionnaire-54, Krupp Fatigue Severity Scale; quality of life, Functional Assessment of Chronic Illness Therapy-Fatigue, and Chalder Fatigue Severity Questionnaire.

**Table 2:** General characteristics of studies in the final analysis (n = 12).

Study (author, year)	Participants (n)		Design	Duration	Disease	Treatment		Type of ginseng	Fatigue evaluation tools	Outcome
	Control	Intervention				Control	Intervention (ginseng)			
Barton et al, 2010^[15]^	Female: 45 Male: 24	Female: 141 Male: 72	RCT	8 wk	Cancer	Placebo	750, 1000, or 2000 mg/d	Wisconsin ginseng	BFI, SF-36	BFI (AUC): 460 (placebo), 467 (750 mg/d), 480 (1000 mg/d), and 551 (2000 mg/d), *P* = .08; SF-36: 7.3 (placebo), 7.8 (750 mg/d), 10.5 (1000 mg/d), and 14.6 (2000 mg/d), *P* = .08.
Kim et al 2011^[16]^	Female: 44 Male: 3		RCT	6 wk	Multiple sclerosis	Placebo	100, 200, 400 mg/d	American ginseng	FSS, RDFS, MFIS	FSS: baseline (5.7 ± 0.98), placebo (5.5 ± 1.3), ginseng (5.5 ± 1.3), *P* = .48; MFIS: baseline (47.0 ± 15.0), placebo (43.7 ± 16.7), ginseng (42.7 ± 15.7), *P* = .23; RDFS: baseline (4.4 ± 1.6), placebo (4.2 ± 1.8), ginseng (4.2 ± 1.6), *P* = .08; RDFS with mixed modeling: baseline (4.2 ± 0.3), placebo (4.1 ± 0.3), ginseng (3.9 ± 0.3), *P* = .0031.
Barton et al, 2013^[17]^	Female: 138 Male: 33	Female: 128 Male: 42	RCT	8 wk	Cancer	Placebo	2000 mg/d	Wisconsin ginseng	MFSI-SF	Change score of MFSI-SF vs baseline: placebo (10.3), ginseng (20), *P* = .003.
Kim et al, 2013^[18]^	Female: 24 Male: 6	Female: 45 Male: 15	RCT	4 wk	Idiopathic chronic fatigue	Placebo	1000 or 2000 mg/d	Panax ginseng C.A. Meyer	NRS, VAS	NRS: placebo (18.8 ± 2.9), 1 g of ginseng (15.1 ± 6.5), 2 g of ginseng (13.8 ± 6.2), *P* < .01; VAS: placebo (5.8 ± 1.3), 2 g of ginseng (4.4 ± 1.8), *P* < .01.
Etemadifar et al, 2013^[19]^	Female: 26 Male: 0	Female: 26 Male: 0	RCT	3 mo	multiple sclerosis	Placebo	250 mg/d	Korean ginseng	MFIS	Placebo: baseline (22.23 ± 13.21), after intervention (23.69 ± 12.94), *P* = .68; ginseng: baseline (31.69 ± 14.9), after intervention (23.65 ± 12.8), *P* = .042. Statistically significant difference in the change of fatigue scores between 2 groups (*P* = 0.46).
Braz et al 2013^[20]^	Female: 13 Male: 0	Female: 13 Male: 0	RCT	12 wk	Fibromyalgia	Placebo	100 mg/d	Panax ginseng	VAS	VAS fatigue: no statistically significant differences in improvement between the mean scores of the 3 groups (*F* (2) = 0.9; *P* > .05).
Hong et al, 2016^21^	Female: 16 Male: 50		RCT	3 wk	Nonalcoholic fatty liver	Placebo	3000 mg/d	Korean red ginseng	KFSS	Placebo: baseline (29.8 ± 14.1), after intervention (25.9 ± 12.5), *P* = .024; ginseng: baseline (33.0 ± 11.6), after intervention (24.3 ± 8.4), *P* < .001. No statistically significant difference in the change of fatigue scores between 2 groups (*P* = .221).
Hee et al, 2017^[22]^	Female: 15 Male: 0	Female: 15 Male: 0	RCT	12 wk	Epithelial ovarian cancer	Placebo	3000 mg/d	Red ginseng	BFI	Placebo: baseline (19.25 ± 10.33), after intervention (11.38 ± 11.84), *P* = .084; ginseng: baseline (23.24 ± 17.79), after intervention (14.29 ± 17.59), *P* = .014.
Pourmohamadi et al, 2017^[23]^	Female: 29 Male: 31	Female: 26 Male: 28	RCT	30 d	Nonmetastatic cancer	Placebo	100 mg/d	Panax ginseng	BEK test	Placebo: baseline (good [13], depression [34] and severe [13]), after intervention (good [11], depression [39] and severe [10]), *P* = .887; ginseng: baseline (good [13], depression [30] and severe [9]), after intervention (good [44], depression [10] and severe [0]), *P* < .0001.
Yennurajalingam et al, 2018^[24]^	Female: 24 Male: 40	Female: 29 Male: 34	RCT	4 wk	Advanced cancer	Placebo	400 mg twice/d	Panax ginseng C.A. Meyer	FACIT-F	The mean (SD) FACIT-F subscale scores at baseline, day 15, and day 29 were 22.4 (10.1), 29.9 (10.6), and 30.1 (11.6) for ginseng (*P* < .001), and 24.0 (9.4), 30.0 (10.1), and 30.4 (11.5) for placebo (*P* < .001). Mean (SD) improvement in the FACIT-F subscale at day 29 was not significantly different in the PG than in the placebo group (7.5 [12.7] vs 6.5 [9.9]; *P* = .67).
Sung et al, 2019^25^	Female: 18 Male: 5	Female: 15 Male: 9	RCT	10 wk	Moderated chronic fatigue	Placebo	3000 mg/d	Korean red ginseng	VAS, FSS, CFSQ	Each group had significant reductions in the fatigue VAS, FSS and CFSQ from baseline (week 0, visit 2) to 6 or 10 wk, but no statistically significant difference in the change of fatigue scores between 2 groups
Kim et al, 2020^[26]^	Female: 80 Male: 123	Female: 82 Male: 124	RCT	16 wk	Colorectal cancer	Placebo	500 mg twice/d	Korean red ginseng	BFI	For the mean AUC change from baseline of BFI over 16 wk in the full analysis set, the ginseng group showed a benefit compared to the placebo group for all questions.

AUC=Area under the Curve, BFI = Brief Fatigue Inventory, CFSQ = Chalder Fatigue Severity Questionnaire, FACIT-F = Functional Assessment of Chronic Illness Therapy-Fatigue, FSS = Fatigue Severity Scale, KFSS = Krupp Fatigue Severity Scale, MFIS = Modified Fatigue Impact Scale, MFSI-SF = Multidimensional Fatigue Symptom Inventory-Short Form, NRS = numerical rating scale, QOL = quality of life, RCT = randomized controlled trial, RDFS = Real-time Digital Fatigue Score, SD = standard deviation, SF-36 = Short Form 36, VAS = visual analog scale.

### 3.4. Efficacy of ginseng supplements on disease-related fatigue reduction

**Figure 4.**
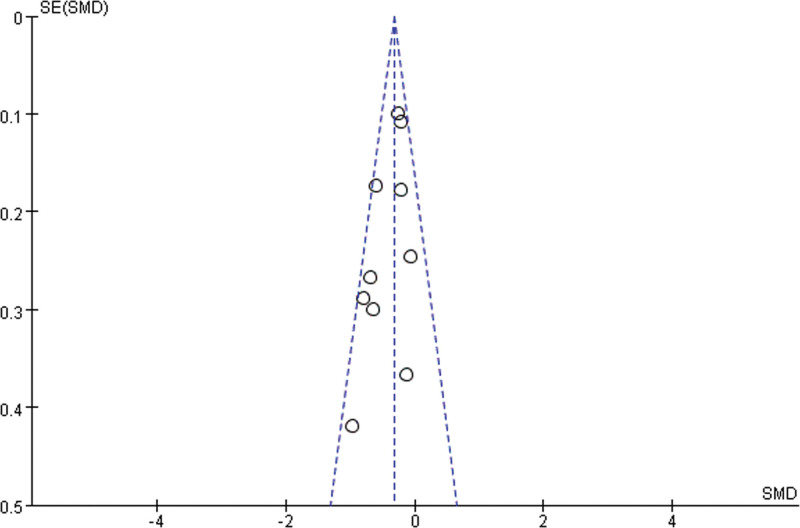
Funnel plot for the effect of ginseng supplements on the disease-related fatigue. SE = standard error, SMD = standardized mean difference.

**Figure 5.**
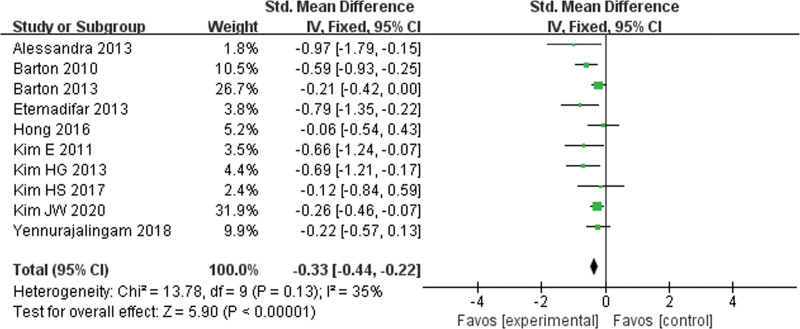
Forrest plot for the effect of ginseng supplements on the disease-related fatigue.

We investigated the efficacy of ginseng supplements on disease-related fatigue reduction, but some included trials were descriptive for the results of fatigue assessment, such as Pourmohamadi et al ^[23]^ and Sung et al^[25]^; so we excluded these 2 trials in meta-analysis. In addition, ginseng interventions in some trials have multiple subgroups, and we only included the data in highest dose of ginseng supplement, such as Barton et al,^[15]^ Kim et al,^[16]^ and Kim et al.^[18]^ As shown in Figure 4, the funnel chart analysis of 10 studies showed the left-right symmetry, which indicated that there is no publication bias. As shown in Figure 5, there is no statistical heterogeneity among the 10 studies (I^2^ = 35%, *P* = .13). Analysis using a fixed-quantity effect model showed that there were statistical differences between the experimental group and the control group (SMD = –0.33, 95% CI = –0.44 to –0.22).

## 4. Discussion

In recent years, domestic and foreign scholars have conducted pharmacological studies, clinical observations, and epidemiological investigations on the effective ingredients of ginseng, and the results have confirmed that ginseng has improved microcirculation, improved tissue antihypoxia ability, and inhibited platelet aggregation, antitumor, and antiaging, antiradiation, antifatigue, and other biological activities.^[27, 28]^ In fatigue, previous studies have found that ginseng can improve thinking and physical activity.^[29, 30]^ However, large samples of clinical research data are insufficient.

In this meta-analysis of RCTs, we aim to investigate the efficacy of ginseng supplements on disease-related fatigue reduction based on 12 RCTs, and we found that ginseng supplements had a statistically significant efficacy on disease-related fatigue reduction (SMD = 0.33, 95% CI = 0.44–0.22) based on Cohen rule of thumb.^[31]^ However, it should be noted that although 12 RCTs were included in final analysis, 2 studies (Pourmohamadi et al^[23]^ and Sung et al^[25]^) were excluded in final meta-analysis in Review Manager 5.3 software, because the evaluation of fatigue in these 2 studies is count data. In addition, ginseng interventions in some trials have multiple subgroups, and we only included the data in highest dose of ginseng supplement, such as Barton et al,^[15]^ Kim et al,^[16]^ and Kim et al.^[18]^ Taken together, the results of our meta-analysis that high dose of ginseng supplements had a statistically significant efficacy on disease-related fatigue reduction.

The antifatigue effect of ginseng is time and dose dependent. Barton et al^[15]^ found that the area under the curve analysis of activity interference from BFI was 460, 467, 480, and 551 in the placebo group, 750 mg/d group, 1000 mg/d group, and 2000 mg/d group, respectively. Similarly, change from baseline in the vitality subscale of the Short Form 36 (SF-36) was 7.3, 7.8, 10.5, and 14.6, respectively. Moreover, the results of low-dose ginseng supplement intervention experiments also showed that it is ineffective against fatigue, such as in Kim et al,^[16]^ Braz et al,^[20]^ and Yennurajalingam et al.^[24]^ However, the type of ginseng was also important. The low dose of Asian ginseng (250 mg/d in Etemadifar et al^[19]^ and 100 mg/d in Pourmohamadi et al^[23]^) significantly reduced fatigue in patients, which was compared with placebo group.

Lots of possible biological mechanisms for the efficacy of ginseng supplements on fatigue have been announced. The feeling of fatigue is thought to be related to excessive consumption of muscle sugar and blood sugar, that is, when muscle glycogen and blood sugar uptake speed is greater than the speed of liver glycogen decomposition into the blood, it will cause a decrease in blood sugar, a decline in work ability, and induce fatigue.^[32,33]^ Fortunately, Yokozawa et al^[34]^ found that the content of hepatic glucose-6-phosphate dehydrogenase activity increased rapidly 2 to 4 hours after administration of ginseng extract. Furthermore, reducing lactic acid accumulation is another important biological mechanism of ginseng to fight fatigue. The increase of lactic acid in the human body leads to an increase in the concentration of hydrogen ions, which interferes with the physiological activity of calcium ions, affects the coupling process of muscle excitation/contraction, reduces muscle contraction strength, and induces fatigue.^[35,36]^ Previous studies have found that ginseng intervention can increase lactic acid metabolism by increasing oxygen intake and lactate dehydrogenase activity in mice.^[37]^

In our meta-analysis, our results are consistent with animal and in vitro studies. However, our meta-analysis has limitations: only 1298 participant, the sample size is small, and some studies are partially added. Nonetheless, ginseng is beneficial in reducing disease-related fatigue based on our study.

## Author contributions

Mingjun Liua and Zhihong Wang designed and funded this study; Jianxun Zhua and Xiaoru Xua completed the writing and data analysis of this study; Jianxun Zhua, Xiaoru Xua, Xin Zhanga, Yue Zhuoa, Shaotao Chena, and Chongwen Zhonga assisted in literature review, data extraction and analysis.
